# Sleep duration, insomnia and cognitive performance in the Elsa-Brasil cohort: a cross-sectional analysis

**DOI:** 10.1590/1980-549720240006

**Published:** 2024-02-05

**Authors:** Tamiris Amanda Rezende, Luana Giatti, Sara Teles de Menezes, Rosane Harter Griep, Pricila Cristina Correa Ribeiro, Sandhi Maria Barreto

**Affiliations:** I Universidade Federal de Minas Gerais, Posgraduate Program in Public Health, Medical School – Belo Horizonte (MG), Brazil.; II Universidade Federal de Minas Gerais, Medical School and Clinical Hospital/EBSERH – Belo Horizonte (MG), Brazil.; III Instituto Oswaldo Cruz, Laboratory of Health and Environment Education – Rio de Janeiro (RJ), Brazil.; IV Universidade Federal de Minas Gerais, Department of Psychology, Faculty of Philosophy and Human Sciences – Belo Horizonte (MG), Brazil.

**Keywords:** Sleep duration, Insomnia, Cognitive performance, Age, Duração do sono, Insônia, Desempenho cognitivo, Idade

## Abstract

**Objective:**

To investigate the single and combined associations between sleep disturbances (sleep duration, insomnia symptoms in the last 30 nights, and daytime tiredness) and performance in cognitive tests.

**Methods:**

Cross-sectional analysis of data from visit 2 (2012–2014) of the Longitudinal Study of Adult Health from a cohort of active and retired civil servants from six Brazilian capitals. Polynomial regression with quadratic term and multiple linear regression models were performed to assess single and combined associations between sleep disturbances and memory performance, fluency, executive functions, and global cognition.

**Results:**

A total of 7,248 participants were included, with a mean age of 62.7 years (standard deviation [SD]=5.9), and 55.2% were women. Inverted U-shaped associations were observed between sleep duration and performance on all cognitive abilities, suggesting that durations shorter or longer than seven hours are associated with worse performance, regardless of age. Reported insomnia was associated with worse executive function (β: -0.08; 95% confidence interval [CI]: -0.15 to -0.01), and the magnitudes of associations were higher for individuals with insomnia at two or more moments (β: -0.12; 95%CI -0.19 to -0.05) or, especially, insomnia combined with short sleep (β: -0.18; 95%CI -0.24 to -0.11). Insomnia in two or more periods was also associated with lower memory and global cognition. There was no association between any sleep disturbance tested and verbal fluency. Isolated daytime tiredness was not associated with performance in the evaluated tests.

**Conclusion:**

The results suggest that extreme sleep durations are detrimental to almost all cognitive abilities investigated, whereas insomnia appears to affect more severely the executive function.

## RESUMO

**Objetivo:**

Investigar a associação isolada e combinada entre distúrbios do sono (duração do sono, sintomas de insônia nas últimas 30 noites e cansaço diurno) e desempenho em testes cognitivos.

**Métodos:**

Análise transversal dos dados da visita 2 (2012–2014) do Estudo Longitudinal de Saúde do Adulto de coorte de servidores públicos ativos e aposentados de seis capitais brasileiras. Regressão polinomial com termo quadrático e modelos de regressão linear múltipla foram realizados para avaliar associações isoladas e combinadas entre distúrbios do sono e desempenho na memória, fluência, funções executivas e cognição global.

**Resultados:**

Foram incluídos um total de 7.248 participantes, com média etária de 62,7 anos (desvio padrão [DP]=5,9), sendo 55,2% mulheres. Associações em forma de U invertido foram observadas entre duração do sono e desempenho em todas as habilidades cognitivas, sugerindo que durações menores ou maiores que sete horas estão associadas ao pior desempenho, independentemente da idade. O relato de insônia foi associado à pior função executiva (β: -0.08; IC95% -0.15 a -0.01), sendo as magnitudes das associações maiores para indivíduos com insônia em dois ou mais momentos (β: -0.12; intervalo de confiança [IC]95% -0.19 a -0.05) ou, especialmente, insônia combinada com sono curto (β: -0.18; IC95% -0.24 a -0.11). Insônia em dois ou mais períodos também foi associada à menor memória e cognição global. Não houve associação entre qualquer distúrbio do sono testado e fluência verbal. Cansaço diurno isolado não foi associado ao desempenho nos testes avaliados.

**Conclusão:**

Os resultados sugerem que a duração extrema do sono é prejudicial para quase todas as funções cognitivas investigadas, enquanto a insônia parece afetar mais fortemente a função executiva.

## INTRODUCTION

Indispensable for cardiometabolic health^1^, sleep is also essential for mental health and cognitive performance^2^, especially for memory consolidation and executive functions^3^. Sleep disturbances, such as extreme durations and insomnia, have been associated with worse global cognitive performance^4^, memory^5^, executive function^6^, and fluency^7^, and predict increased risk of dementia, including Alzheimer’s disease^8^. Besides, aging is associated with shorter sleep duration and impaired sleep quality due to changes in the regular non-rapid eye movement (NREM) and REM cycles^9^. Therefore, investigating the association between sleep disorders and cognition in healthy middle-aged and older adults is essential to determine whether they act as a risk factor early in adulthood or are prodromal dementia symptoms at the end of life^10^.

Findings from a study including middle-aged and older adults indicated an association of short (<7 hours) and long (≥8 hours) sleep duration with worse performance in abilities, such as reasoning, basic reaction time, short-term numerical memory, visual memory, and prospective memory^5^. Also, a faster decline in the global cognitive score in individuals with short (≤4 hours) and long sleep duration (≥10 hours) was reported^11^. These results suggest inverted U-shaped association between sleep duration and decline in cognitive abilities. Notably, the cutoff points for long and short sleep duration vary among studies, impairing comparisons.

In turn, associations between insomnia and cognitive abilities need to be more consistent. A representative cohort of the United States population aged 50 years and over showed that early awakening was associated with global cognitive decline after a 10-year follow-up^4^, but another representative sample of American older adults did not find this longitudinal relationship^12^. Furthermore, in the literature, we find cross-sectional associations between insomnia and worse^13^ and better^5^ memory performance.

It appears that primary insomnia, characterized as difficulty in initiating or maintaining sleep with resulting impaired daytime functioning such as tiredness or drowsiness^14^, combined with short sleep duration, is more harmful to cognitive performance^14^, especially for abilities linked to higher-order processes, like working memory and attention shift^15^. However, the different characteristics of sleep (insomnia and sleep duration) are commonly not analyzed separately in the same study, or the studies do not include adjustments for factors related to sleep, such as depression and the use of sleep medications^16^, making it difficult to point out which characteristics affect cognitive performance and, consequently, the occurrence of dementia^10^.

This study aimed to investigate isolated and combined associations between sleep disturbances (sleep duration, insomnia symptoms in the last 30 nights, and daytime tiredness) and performance in cognitive tests. We hypothesized a curvilinear relation between sleep duration and cognition, and that extreme durations, as well as reported insomnia, are associated with worse cognitive performance, regardless of daytime fatigue, in different abilities, especially memory and executive function. In addition, we hypothesized that insomnia with short sleep duration is more strongly associated with all cognitive abilities assessed than each of these disorders separately.

## METHODS

### Study design and sample

A cross-sectional analysis was performed with data from visit 2 (2012-2014) of the Longitudinal Study of Adult Health (*Estudo Longitudinal de Saúde do Adulto* – ELSA-Brasil), from a cohort of 15,105 civil servants from higher education and research institutions, in six Brazilian capitals, started in 2008 until 2010^17^. Blood collection for biochemical tests, weight, height, and blood pressure measurements was obtained after fasting (10 to 14 hours), following standardized techniques performed by trained and certified professionals^18^. The research ethics committees of the institutions involved approved the study. All participants signed an Informed Consent Form. The complete description of the inclusions and exclusions is detailed in the Supplementary Figure 1.

### Sleep assessments

The explanatory variables were sleep duration, frequency of insomnia symptoms in the last 30 nights, insomnia, daytime tiredness, insomnia and daytime tiredness, and insomnia and sleep duration. The test-retest reliability of insomnia symptoms and self-reported sleep duration questions was assessed in a subsample of 205 participants randomly recruited, considering an interval of 7–14 days between the interviews. The intraclass correlation coefficient (ICC) for sleep duration was good (ICC 0.761; 95%CI 0.685 to 0.819), and substantial agreement for insomnia was observed (Kappa 0.759; 95%CI 0.651 to 0.867)^19^.

Sleep duration (hours): It was measured by the question "How many hours, on average, do you sleep in a regular night’s sleep?". It was used as a continuous and categorical variable grouped into short sleep duration (<6 hours), regular sleep duration (≥6 hours ≤8 hours), and long sleep duration (>8 hours). These cutoff points were based on the results of the polynomial regression analysis using age as the quadratic term, in addition to the recommendation by the National Sleep Foundation^20^, and adoption in previous studies^19–21^.

Insomnia symptoms in the last 30 nights: Its frequency was obtained by the answers to the questions:

- How often, in the last 30 nights, did you experience difficulty falling asleep?";
- How often, in the last 30 nights, did you wake up during sleep and experience difficulty falling asleep again?";
- How often, in the last 30 nights, did you wake up before the desired time and could not fall asleep again?".

According to five possible answer options, not having insomnia was considered "never, rarely, and sometimes", and presence of insomnia as "almost always and always". Finally, the frequency of insomnia symptoms was categorized into three groups^19^:

- without insomnia;
- insomnia at one night period (onset or middle, nocturnal awakenings, or end); and
- insomnia at two or more night periods (start/middle, start/end, middle/end).

Insomnia (no/yes): It was considered insomnia symptoms at any frequency as yes in order to verify the specific effect of insomnia symptoms in the last 30 nights, regardless of the presence of daytime tiredness or sleep duration.

Daytime tiredness: The answer to the following question was used to assess daytime tiredness: "Do you often feel tired, fatigued or sleepy during the day?", with "no/yes" response options.

Insomnia and daytime tiredness: The combination of insomnia variable (no/yes) and daytime tiredness (no/yes) resulted in four categories:

- without insomnia and without daytime tiredness;
- only insomnia;
- only daytime tiredness; and
- insomnia and daytime tiredness.

Insomnia and sleep duration: The combination of the insomnia variable (no/yes) and the sleep duration categories generated five groups:

- without insomnia and regular sleep (≥6 hours ≤ 8 hours; n=4,297);
- without insomnia and short sleep duration (<6 hours; n=652);
- without insomnia and long sleep duration (>8 hours; n=273);
- insomnia and regular sleep duration (n=935); and
- insomnia and short sleep duration (n=807)^19^.

Due to the small number of individuals, the category of insomnia and long sleep duration (n=45) was not included in the combined analysis between reports of insomnia and sleep duration.

### Cognitive assessments

Three cognitive tests (memory, verbal fluency, and trail B) and the global cognition score were evaluated, and z-scores were obtained by subtracting the mean from the test score and dividing the result by its standard deviation. Except for Phonemic Verbal Fluency, all tests are part of the neuropsychological battery Consortium to Establish a Registry for Alzheimer’s Disease (CERAD), validated for the Brazilian older adult population^22^. The baseline reliability of the cognitive tests, as assessed by the intraclass correlation coefficient, ranged from moderate to nearly perfect^23^.

Memory test: The total score ranged from 0 to 50 correct words on learning, recall, and word recognition tests. These tests comprised ten unrelated words presented every two seconds, on cards with large letters, and in a different order in each of the three learning trials, with immediate recall. After five minutes, retention and recall were tested by a free recall and recognition of the previous ten words that were mixed with ten others distracting words^23^.

Verbal fluency test: It consisted of asking participants to say as many words as possible related to a specific category such as flora (semantic test), starting with the letter A (phonemic test), in one minute^23^. The total score ranged from 0 to 68 correct words obtained in those tests. This test’s critical component is the language processing assessment^24^.

Trail test (part B): This test assessed executive function, attention, concentration, and psychomotor speed^25^. First, the participants performed the Trail A test as training, and only upon completion of Trail A, they were asked to perform the Trail B test. Thus, those who failed the Trail A test did not perform Trail B. The participants were instructed to draw a line connecting letters and numbers, alternating numbers in ascending order with letters in alphabetical order (1, A, 2, B, 3, C, etc.). The line should be done as quickly as possible without lifting the tip of the pencil from the sheet. Supervisors were instructed to point out errors^23^. The total score was the time in seconds to complete Trail B (from 34 to 1,853 seconds). Trail A test runtime was not recorded. For participants who did not complete the Trail B test (n=302), the time to complete the test was estimated as the maximum time spent by individuals of the same sex, age, and schooling level, plus one second, considering these variables were shown to be strong predictors for completion of the test^26^. For statistical analysis, the score was inverted (1 ÷ test time in seconds) to be in the same direction as the other tests, i.e., with higher values indicating better performance.

Global cognitive score: The global cognitive factor (g factor) was obtained from factor analysis of the standardized scores (z-score) of each cognitive test mentioned above. The g factor was the first factor and explained 65% of the total variance of the cognitive tests. This proportion is a typical variance value explained by the g factor^27^.

### Covariates

Confounding variables were those known to be associated simultaneously with sleep disturbances and cognitive performance in the literature on the subject.

Sociodemographic: sex, age (years), and schooling level (graduate level, complete higher education, complete secondary education, complete primary education, incomplete primary education).

Health behaviors: smoking (never smoked, former smoker, smoker); physical activity, measured by the long version of the International Leisure Physical Activity Questionnaire and grouped according to metabolic equivalent of the task (MET) into weak (<600 MET min/week), moderate (600±3,000 MET min/week) and vigorous (≥3,000 MET min/week)^28^; alcohol consumption (moderate, does not use, excessive), measured by weekly consumption (grams), being moderate if <210 g/week for men and <140 g/week for women, and excessive if ≥210 g/week for men and ≥140 g/week for women^29^.

Health conditions: depression (no/yes) measured by the Portuguese version^30^ of the Clinical Interview Schedule-Revised (CIS-R)^31^; continuous body mass index (BMI) (kg/m^2^); hypothyroidism (no/yes) defined by laboratory findings (TSH >4.78 μU/mL and free T4 <0.89 ng/dL) or use of levothyroxine; the number of comorbidities (none, one, two or more) considering the presence of the following diseases: diabetes mellitus (defined by a medical diagnosis or any of the following criteria: use of antidiabetic drugs, or fasting glucose ≥126 mg/dL, or glucose test tolerance ≥200 mg/dL, or glycated hemoglobin ≥6.5%), hypertension (use of antihypertensive drugs and/or systolic blood pressure [SBP] ≥140 mmHg and/or diastolic BP [DBP] ≥90 mmHg), medical diagnosis report of cardiovascular disease, cancer, and chronic obstructive pulmonary disease (no/yes); and use of the benzodiazepine or non-benzodiazepine hypnotics (no/yes)^32^.

### Statistical analysis

Categorical variables were described as proportions, and continuous variables as mean and standard deviation or median and interquartile ranges, whenever appropriate.

We first analyzed the association between sleep duration and cognitive performance, hypothesizing the existence of a curvilinear relation between these variables, using a polynomial regression analysis considering age as a quadratic term.

Second, the associations of the explanatory variables with the performance in each cognitive test (outcome) were investigated using multiple linear regression models.

After a raw regression analysis (Model 0), sequential adjustments were made for

- sex, age, schooling (Model 1);
- smoking habit, alcohol consumption, and physical activity (Model 2),
- BMI, number of comorbidities, depression, hypothyroidism, and use of benzodiazepine or non-benzodiazepine hypnotics (Model 3).

The β value indicates how much worse (or better) the exposed individuals perform on average in relation to the reference category. In other words, it compares the average total cognitive scores (in standardized units) of participants in a given exposure category (e.g., sleep "<6 hours sleep") with participants in the reference category (sleep ≥6 hours ≤8 hours).

The linear regression assumptions for the error terms (constant variance, normal distribution, and independence) were verified graphically.

We considered p<0.05 as statistically significant and 95% confidence interval. Analyses were conducted by Stata (Stata Corporation, College Station, Texas, USA), version 14.0, and by R, version 4.0.3.

## RESULTS

A total of 7,248 individuals, corresponding to 51.2% of the participants of visit 2 (n=14,014), and aged ≥55 years performed the cognitive tests. The studied population (n=7,009), after exclusions, had an average age of 62.7 (SD=5.9) years, mostly women (55.2%), and 44.1% had graduate education. On average, participants reported sleeping 6.5 (SD=1.4) hours/night and 25.5% had insomnia for at least one moment (Supplementary Table 1).

After considering all covariates in the analysis, there was a curvilinear statistical association (inverted U) between sleep duration and performance in all assessed abilities (Supplementary Table 2).

**Figure 1:**
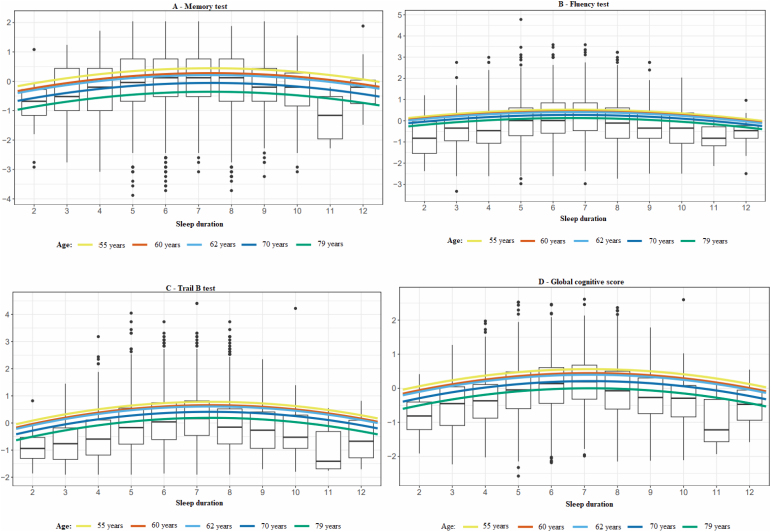
(A to D): Curvilinear association between sleep duration and cognitive performance in (A) memory, (B) verbal fluency, (C) trail B test, and (D) global cognitive score, in different age groups, after all adjustments. ELSA-Brasil (2012–2014). n=7,009.

Figure 1 (A to D) shows the observed values and non-linear estimates (quadratic term) between sleep duration and cognitive performance in different age groups. Overall, cognitive performance improved until sleep duration reached approximately seven hours and declined afterward. We observed lower mean scores on all abilities with advancing age, but the inverted curvilinear relation between sleep duration and cognitive performance remained unchanged at around seven hours.

Considering the report of frequency of insomnia symptoms in the last 30 nights, we observed in the adjusted analysis, that the report of insomnia at one night period (beginning, middle, or end) was associated with worse performance only in executive function (β: -0.08; 95%CI -0.15 to -0.01), when compared to those who did not report insomnia. The participants who reported insomnia in two or three night periods, on the other hand, had lower memory test (β: -0.09; 95%CI -0.16 to -0.01), executive function (β: -0.12; 95%CI -0.19 to -0.05) and global cognition scores (β: -0.07; 95%CI -0.12 to -0.02) than those without insomnia (Table 1).

**Table 1:** Association between frequency of insomnia symptoms in the last 30 nights and performance on cognitive function tests standardized by z-score at visit 2 of ELSA-Brasil (2012–2014). n=7,009.

Cognitive function test (z-score)		Frequency of insomnia symptoms in the last 30 nights		
		Without insomnia	Insomnia at one night period	Insomnia at two or more night periods
			β (95%CI)	β (95%CI)
Memory test		n=6,819		
	Raw model	Ref.	-0.04 (-0.11; 0.03)	-0.18 (-0.25; -0.11)*
	Model 1		-0.03 (-0.10; 0.04)	-0.08 (-0.15; -0.02)†
	Model 2		-0.03 (-0.10; 0.04)	-0.08 (-0.14; -0.01)†
	Model 3		-0.01 (-0.08; 0.06)	-0.09 (-0.16; -0.01)†
Verbal fluency test		n=6,869		
	Raw model	Ref.	-0.04 (-0.11; 0.03)	-0.17 (-0.24; -0.10)*
	Model 1		-0.00 (-0.07; 0.06)	0.01 (-0.05; 0.07)
	Model 2		-0.01 (-0.07; 0.05)	0.01 (-0.05; 0.07)
	Model 3		-0.01 (-0.08; 0.06)	-0.02 (-0.09; 0.05)
Trail B test		n=6,903		
	Raw model	Ref.	-0.14 (-0.21; -0.06)*	-0.36 (-0.43; -0.29)*
	Model 1		-0.08 (-0.14; -0.02)†	-0.14 (-0.20; -0.08)*
	Model 2		-0.09 (-0.15; -0.03)†	-0.14 (-0.19; -0.08)*
	Model 3		-0.08 (-0.15; -0.01)‡	-0.12 (-0.19; -0.05)*
Global cognitive score		n=6,765		
	Raw model	Ref.	-0.06 (-0.12; -0.01)‡	-0.22 (-0.28; -0.17)*
	Model 1		-0.03 (-0.08; 0.01)	-0.06 (-0.10; -0.02)†
	Model 2		-0.04 (-0.08; 0.01)	-0.06 (-0.10; -0.01)†
	Model 3		-0.03 (-0.08; 0.02)	-0.07 (-0.12; -0.02)†

β: coefficients obtained by linear regression; CI: confidence interval.

* p-value ≤0.001

† ≤0.010

‡ ≤0.050.

Model 0: Raw model; Model 1: Model 0 + sex, age, and schooling; Model 2: Model 1 + physical activity, smoking habit, and alcohol consumption; Model 3: Model 2 + body mass index, comorbidities, hypothyroidism, depression, and use of benzodiazepine or non-benzodiazepine hypnotics.

In the final analyses, considering the co-occurrence of insomnia and tiredness (Table 2), we found daytime tiredness alone (without insomnia) was not associated with any of the investigated abilities. However, the report of insomnia without tiredness was associated with poorer performance in executive function (SD=0.09; 95%CI -0.16 to -0.01) below the mean, whereas participants who reported insomnia plus daytime tiredness performed worse in memory test (β: 0.08; 95%CI -0.15 to -0.01), executive function (β: -0.10; 95%CI -0.16 to -0.03), and global cognition scores (β: -0.06; 95%CI -0.11 to -0.01), when compared to individuals without insomnia and without tiredness (Table 2).

**Table 2:** Association of the variable insomnia and daytime tiredness with performance on cognitive function tests standardized by z-score at visit 2 of ELSA-Brasil (2012–2014). n=7,009.

Cognitive function test (z-score)		Insomnia and daytime tiredness			
		Without insomnia and without daytime tiredness	Only insomnia	Only daytime tiredness	Insomnia and daytime tiredness
			β (95%CI)	β (95%CI)	β (95%CI)
Memory test		n=6,819			
	Raw model	Ref.	-0.14(-0.22; -0.06)*	-0.03 (-0.09; 0.03)	-0.12 (-0.19; -0.05)*
	Model 1		-0.03 (-0.10; 0.04)	-0.05 (-0.11; -0.00)†	-0.10 (-0.17; -0.04)*
	Model 2		-0.03 (-0.10; 0.04)	-0.04 (-0.09; 0.01)	-0.09 (-0.15; -0.03)‡
	Model 3		-0.04 (-0.12; 0.04)	-0.05 (-0.10; 0.01)	-0.08 (-0.15; -0.01)†
Verbal fluency test		n=6,869			
	Raw model	Ref.	-0.13(-0.22; -0.05)*	-0.02 (-0.08; 0.03)	-0.10 (-0.17; -0.03)‡
	Model 1		0.17 (-0.05; 0.09)	-0.02 (-0.07; 0.02)	-0.02 (-0.08; 0.04)
	Model 2		0.01 (-0.06; 0.08)	-0.01 (-0.06; 0.04)	-0.01 (-0.06; 0.05)
	Model 3		0.00 (-0.08; 0.07)	-0.01 (-0.07; 0.04)	-0.03 (-0.10; 0.04)
Trail B test		n=6,903			
	Raw model	Ref.	-0.28(-0.36; -0.19)*	0.00 (-0.06; 0.06)	-0.25(-0.32; -0.18)*
	Model 1		-0.10 (-0.16; -0.03)‡	0.01 (-0.04; 0.06)	-0.12(-0.18; -0.06)*
	Model 2		-0.10 (-0.17; -0.04)‡	0.01 (-0.03; 0.06)	-0.11(-0.17; -0.05)*
	Model 3		-0.09 (-0.16; -0.01)‡	0.01 (-0.04; 0.07)	-0.10 (-0.16; -0.03)‡
Global cognitive score		n=6,765			
	Raw model	Ref.	-0.18(-0.24; -0.11)*	-0.02 (-0.06; 0.03)	-0.14(-0.20; -0.09)*
	Model 1		-0.03 (-0.08; 0.02)	-0.02 (-0.05; 0.02)	-0.07(-0.11; -0.03)*
	Model 2		-0.04 (-0.09; 0.01)	-0.01 (-0.04; 0.03)	-0.06 (-0.10; -0.01)‡
	Model 3		-0.04 (-0.10; 0.01)	-0.01 (-0.05; 0.03)	-0.06 (-0.11; -0.01)‡

β: coefficients obtained by linear regression; CI: confidence interval.

* p-value ≤0.001

† ≤0.050

‡ ≤0.010.

Model 0: Raw model; Model 1: Model 0 + sex, age, and schooling; Model 2: Model 1 + physical activity, smoking habit, and alcohol consumption; Model 3: Model 2 + body mass index, comorbidities, hypothyroidism, depression, use of benzodiazepine or non-benzodiazepine hypnotics, and sleep duration.

The analyses considering insomnia and sleep duration simultaneously (Table 3) showed, after all adjustments, that individuals without insomnia but with long sleep duration had worse performance in memory test (β: -0.12; 95%CI -0.24 to -0.00), whereas participants without insomnia but with short sleep duration had worse performance in executive function (β: -0.13; 95%CI -0.20 to -0.06) and global cognition scores (β: -0.07; 95%CI -0.12 to -0.01). Individuals who reported insomnia and short sleep duration performed worse on memory test (β: -0.13; 95%CI -0.20 to -0.05), executive function (β: -0.18; 95%CI -0.24 to -0.11), and global cognition scores (β: -0.10; 95%CI -0.15; to -0.05) than participants without insomnia and with regular sleep duration.

## DISCUSSION

In this study, including middle-aged and older adults, inverted U-shaped associations were observed between sleep duration and performance in memory, fluency, trail B, and global cognition tests, indicating that lesser and longer sleep durations of about seven hours were associated with worse cognitive performance, more markedly in memory, executive function, and global cognition. There was no association between any sleep disturbance tested and verbal fluency, suggesting that language processing ability may be the least affected by these exposures. Executive function was associated with insomnia without and with daytime tiredness, as well as with insomnia in one or two or more moments, indicating that it might be the cognitive ability mostly affected by insomnia.

Among the examined tests, memory seems to be the most shaken by sleep duration since both short and long durations were associated with worse performance in this test, corroborating evidence of the role of sleep duration in memory consolidation^3^. Cross-sectional analysis with individuals between 65 and 85 years old also suggests an inverted U-shaped association between short (≤6 hours) and long (≥9 hours) sleep duration and skills such as memory and executive function^21^. Considering that individuals with amnestic mild cognitive impairment are at increased risk of dementia^33^, as well as those with persistent duration of ≤6 hours at 50, 60, and 70 years^34^, individuals with extreme sleep durations deserve attention to prevent continued adverse effects on memory. It is possible that short sleep duration combined with insomnia may be more harmful for higher-level cognitive abilities, as the magnitude of the associations is stronger for this group when compared to the other combinations of sleep duration and insomnia variables.

Our results endorse the impact of insomnia on cognition, except for verbal fluency tests, suggesting that its effect may be worse when combined with short sleep or when insomnia occurs in more than one moment. Daytime tiredness did not appear to affect cognitive test performance on its own, and its combination with insomnia made little difference in cognitive performance, vis-à-vis the magnitude of the associations seen with insomnia only.

A cross-sectional study also found that insomnia added to short objective sleep (<6 hours) is associated with impairments in higher-level abilities (executive attentional control)^35^. Similarly, another cross-sectional association between insomnia (initial and intermediate) and poor memory test^36^ was observed in individuals aged >45 years. Insomnia has also been cross-sectionally associated with worse memory performance^13^ in individuals with a mean age of 44.4 years (SD=11.5). A meta-analysis of 24 cross-sectional studies showed a relationship between primary insomnia and worse executive functioning^37^. In addition, in a 5-year longitudinal study of older adults (mean age 71.9 years), polysomnographic insomnia (wakefulness after sleep) increased the chances of global cognitive decline^12^, while earlier awakening was related to global cognitive impairment in subjects with a mean age of 67.5 years over ten years^4^. Thus, the poorer performance on memory and executive function tests among individuals who reported insomnia, as shown in this study, supports arguments about the potential effect of insomnia on complex tasks, such as attention shift, cognitive flexibility, rather than simple tasks, such as attention basic^37^.

Some mechanisms may explain these associations. Individuals with insomnia and poor performance in executive functions presented volume changes in the cortical and subcortical gray matter, including areas involved in Alzheimer’s disease, as well as lesser white matter diffusion, indicating that neuroinflammation mediates this relation^9^. It is further suggested that the association between insomnia and gray matter volume is modulated by the apolipoprotein E (APOE) -ε4 status^6^. Decreased serum levels of brain-derived neurotrophic factor (BDNF) were found in individuals with primary insomnia and objectively measured short sleep duration (<6 hours) and it was correlated with poorer cognitive performance in different abilities, indicating that cognitive impairment may be explained by a low concentration of BDNF^14^. Short duration (≤6 hours) sleep was associated with higher Aβload, while normal (7–8 hours) and long (≥9 hours) duration was not^21^.

The strengths of this study include methodological rigor in data collection, quality assurance control, a large sample size, and adjustment for a wide range of confounding factors. Furthermore, the neuropsychological test battery was standardized and validated for the Brazilian population^38^.

Limitations include the assessment of sleep disturbances by self-report, which can compromise accuracy. However, these measures are more accessible and easier to apply to large epidemiological studies. The cross-sectional design precludes establishing a temporal relation between sleep disorders and cognitive performance. We adjusted the results for using hypnotics instead of classifying the users as having insomnia, which may have underestimated the associations found. The existence of obstructive sleep apnea, associated with cardiometabolic risk and dementia, was not assessed^39^.

Based on a large sample of middle-aged and older adults, the results indicate a curvilinear and continuous relation between extreme sleep durations, greater or less than approximately seven hours, and poorer cognitive performance in different abilities, in addition to global cognition, as we hypothesized. Unlike our hypothesis, reported insomnia was not associated with verbal fluency but with worse performance in executive function, as we presumed, being stronger the association in those who reported insomnia and short duration of sleep.
