# Supplementary material for: Sleep duration, insomnia and cognitive performance in the Elsa-Brasil cohort: a cross-sectional analysis
Source: Rev Bras Epidemiol. 2024 Feb 5;27:e240006. doi: 10.1590/1980-549720240006 (PMC10846421; doi:10.1590/1980-549720240006)

SUPPLEMENTARY MATERIAL

Supplementary Table 1. Sociodemographic, behavioral, and health characteristics of the study population. ELSA-Brazil (2012-2014). N=7,009.

**Characteristics**	**Visit 2**<br>**% / mean (SD) or median (25% - 75%)**
**Age, y**	62,7 (5,9)
**Sex, women**	55,2
**Schooling**	
Graduate level	44,1
Complete higher education	11,3
Complete secondary education	28,4
Complete primary education	8,6
Incomplete primary education	7,6
**Smoking**	
Never smoker	51,8
Former smoker	38,0
Smoker	10,2
**Consuption of alcoholic beverages**	
Moderate	55,1
Not use	36,6
Excessive	8,3
**Leisure-time physical activity**	
Weak	72,9
Moderate	20,3
Strong	6,8
**Depression**	4,1
**Hypothyroidism**	13,8
**Comorbidities**	
None	35,5
One	38,6
Two or more	25,9
**BMI (Kg/m^2)**	27,6 (4,8)
**Use of benzodiazepines and/or hypnotics**	7,1
**Sleep duration, h**	6,5 (1,4)
**Insomnia and daytime tiredness**	
Without insomnia and without daytime tiredness	51,5
Only insomnia	10,1
Only daytime tiredness	23,0
Insomnia and daytime tiredness	15,4
**Frequency of insomnia symptoms in the last 30 nights**	
Without insomnia	74,5
Insomnia at a time	11,5
Insomnia at two or more times	14,0

**Insomnia and sleep duration**	
Without insomnia and regular sleep	61,3
Without insomnia and short sleep duration	9,3
Without insomnia and long sleep duration	4,0
Insomnia and regular sleep duration	13,3
Insomnia and short sleep duration	11,5
**Memory Test score*** (number of correct words)	37,2 (6,2)
**Verbal Fluency Test**† (number of corret words)	27,9 (8,4)
**Trail B Test**‡ (seconds)	112 (82,0 – 170,0)

Abbreviations: SD, standard deviation; BMI, body mass index.
* Score ranging from 0 to 50 correct words;
† Score ranging from 0 to 68 correct words remembered in a time interval of 1 minute;
‡ Score ranging from 34 to 1853 seconds.

Supplementary Table 2. Association between sleep duration and performance standardized by z score on cognitive function tests at visit 2 of ELSA-Brazil (2012-2014). N=7,009.

	**Sleep duration (hours)**	
**Cognitive Function Test (score z)**	Simple term	Quadratic term
	β (IC95%)	β (IC95%)
**Memory tes**	**N= 6,819**	
Raw Model	0,51 (0,41; 0,61)***	-0,04 (-0,04; -0,03)***
Model 1	0,26 (0,17; 0,35)***	-0,02 (-0,03; -0,01)***
Model 2	0,25 (0,16; 0,34)***	-0,02 (-0,02; -0,01)***
Model 3	0,24 (0,14; 0,34)***	-0,02 (-0,02; -0,01)***
**Verbal Fluency Test**	**N= 6,869**	
Raw Model	0,57 (0,47; 0,67)***	-0,04 (-0,05; -0,03)***
Model 1	0,20 (0,11; 0,19)***	-0,02 (-0,02; -0,01)***
Model 2	0,19 (0,10; 0,28)***	-0,01 (-0,02; -0,01)***
Model 3	0,18 (0,09; 0,28)***	-0,01 (-0,02; -0,01)***
**Trail B Test**	**N= 6,903**	
Raw Model	0,75 (0,65; 0,85)***	-0,05 (-0,06; -0,05)***
Model 1	0,32 (0,23; 0,40)***	-0,02 (-0,03; -0,02)***
Model 2	0,31 (0,22; 0,39)***	-0,02 (-0,03; -0,01)***
Model 3	0,33 (0,24; 0,41)***	-0,02 (-0,03; -0,02)***
**Global Cognitive Score**	**N= 6765**	
Raw Model	0,61 (0,53; 0,69)***	-0,04 (-0,05; -0,04)***
Model 1	0,25 (0,19; 0,32)***	-0,02 (-0,02; -0,01)***
Model 2	0,24 (0,18; 0,31)***	-0,02 (-0,02; -0,01)***
Model 3	0,25 (0,18; 0,32)***	-0,02 (-0,02; -0,01)***

Abbreviations: β, coefficients obtained by Linear Regression; CI, Confidence interval; BMI, Body mass index; p-value *≤0,05; **≤0,01; ***≤0,001.

Model 0 = Raw Model

Model 1 = Model 0 + sex, age, schooling.

Model 2 = Model 1 + physical activity, smoking habit and consumption of alcoholic beverages;

Model 3 = Model 2 + body mass index, comorbidities, hypothyroidism, depression, use of benzodiazepine or non-benzodiazepine hypnotics.

Supplementary Figure 1: Study population flowchart

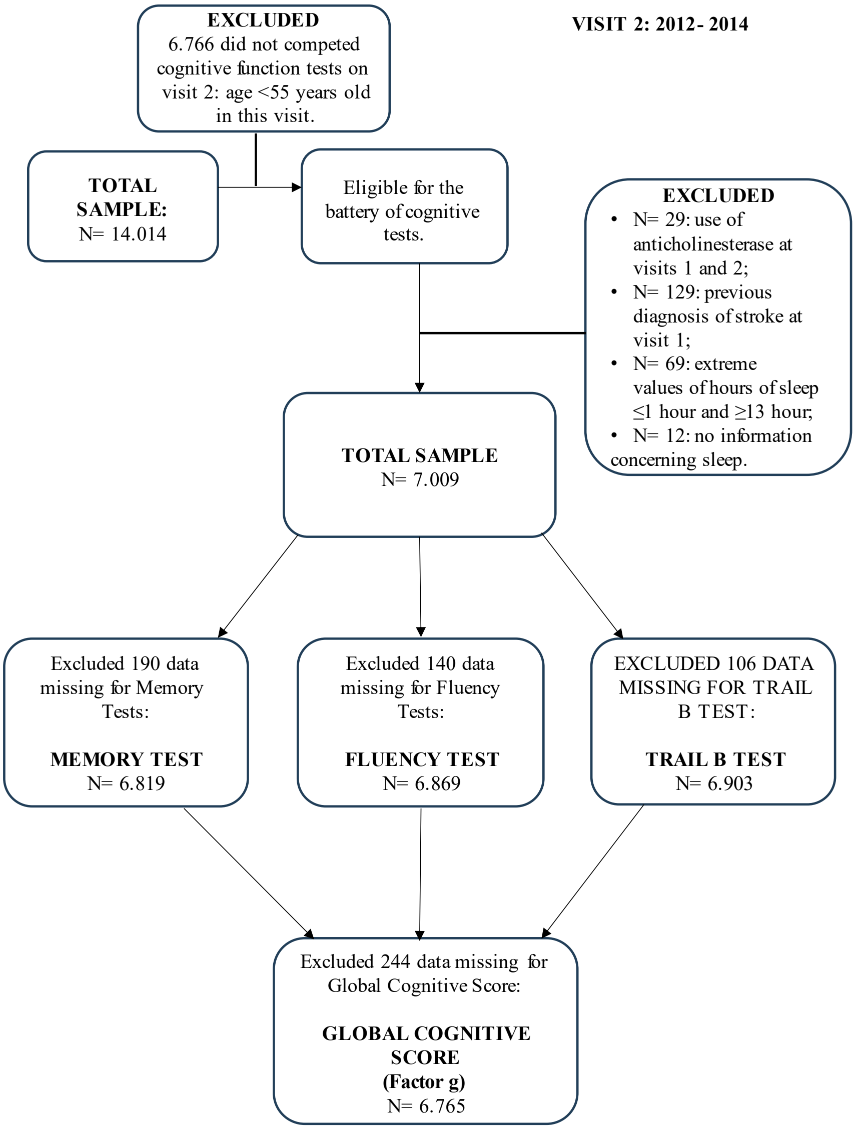

Supplement: Supplementary file 1 [file 1980-5497-rbepid-27-e240006-Suppl01.pdf]
